# Use of Neuroanatomic Knowledge and Neuronavigation System for a Safe Anterior Petrosectomy

**DOI:** 10.3390/brainsci11040488

**Published:** 2021-04-12

**Authors:** Ana Flores-Justa, Sabino Luzzi, Alice Giotta Lucifero, Juan F. Villalonga, Amparo Saenz, José María Santin-Amo, Matias Baldoncini, Alvaro Campero

**Affiliations:** 1 Department of Neurosurgery, Hospital General of Alicante, 3010 Alicante, Spain; annaflores36@gmail.com; 2 Laboratory of Neuroanatomic Microsurgical—LaNeMic-II Division of Anatomy, School of Medicine, University of Buenos Aires, C1053 CABA Buenos Aires, Argentina; drbaldoncinimatias@gmail.com; 3 Neurosurgery Unit, Department of Clinical-Surgical, Diagnostic and Pediatric Sciences, University of Pavia, 27100 Pavia, Italy; alicelucifero@gmail.com; 4 Neurosurgery Unit, Department of Surgical Sciences, Fondazione IRCCS Policlinico San Matteo, 27100 Pavia, Italy; 5 LINT, Facultad de Medicina, Universidad Nacional de Tucumán, T4000 Tucumán, Argentina; jfvillalonga@gmail.com (J.F.V.); amparo_saenz@hotmail.com (A.S.); alvarocampero@yahoo.com.ar (A.C.); 6 Department of Neurological Surgery, Hospital Padilla, T4000 Tucumán, Argentina; 7 Department of Neurological Surgery, Hospital Xeral-Calde, 27004 Lugo, Spain; josemaria.santin@rai.usc.es

**Keywords:** Kawase approach, petroclival area, petrous bone, skull base, transpetrosal approach

## Abstract

Introduction: The petroclival region is among the most challenging anatomical areas to deal with in skull base surgery. Drilling of the anterior part of the petrous bone during the anterior transpetrosal approach involves the risk of injury of the cochlea, superior semicircular canal, internal carotid artery, and internal auditory canal. A thorough understanding of the microneurosurgical anatomy of this region is mandatory to execute the transpetrosal approaches, decreasing the risk of complications. The aim of this study is to describe the anatomical structures of the petroclival region, highlighting the importance of neuronavigation for safe performance of the anterior transpetrosal approach. Methods: Three adult cadaveric human heads were formalin-fixed and injected with colored silicone. They underwent an axial 1 mm slab CT scan, which was used for neuronavigation during the surgical approaches. The anterior petrosectomy was performed with the aid of neuronavigation during the drilling of the petrous bone. The surgical management of a patient harboring a petroclival meningioma, operated on using an anterior transpetrosal approach, was reported as an illustrative case. Results: The anterior petrosectomy was completed accurately with wide exposure of the surgical target without injuring the cochlea and other structures in all three cadaveric specimens. In the surgical case, no approach-related complications occurred, and a gross total resection of the tumor was achieved. Conclusions: Deep knowledge of the location and relationships of the vital elements located within the temporal bone, along with the use of neuronavigation, are the key aspects to perform the anterior transpetrosal approach safely, reducing the risk of complications.

## 1. Introduction

The petroclival region is among the most demanding anatomical areas to deal with in middle and lateral skull base surgery [1,2,3,4,5]. Tumors growing in this area usually displace the cranial nerves, compress the brainstem and cerebellum, and enclose arteries and veins of the posterior circulation. Numerous approaches have been proposed for the treatment of the lesions located in the petroclival region, includuing the anterior transpetrosal and retrosigmoid approach [6,7,8], and presigmoid approaches.

The approach-related morbidity is non-negligible, especially for tranpetrosal ones, including the risk of cerebrospinal fluid fistulas, facial nerve palsy, deafness, facial anesthesia, and vascular injures [7,9,10,11]. This aspect is probably among the main reasons why a number of surgeons are reluctant to use the transpetrosal corridors, recommending alternative approaches for petroclival meningiomas, trigeminal schwannomas, or also basilar apex aneurysms. Nevertheless, the surgical routes directed through the petrous bone are to be considered invasive only for the bone, since their correct execution involves a complete sparing of the neurovascular structures. They permit surgeons to avoid a fixed brain retraction in most cases and also allow for better illumination of the surgical field. Perfect knowledge of the anatomy of the middle cranial fossa is paramount for performing the anterior transpetrosal approaches and for concomitantly reducing the risk of complications.

This study is aimed at overviewing the microneurosurgical anatomy of the petrous region, while also focusing on the importance of neuronavigation to safely and effectively perform the anterior petrosectomy.

## 2. Materials and Methods

The present study was approved by the Internal Review Board (PROT N 20180659017; PROC N: 20187017666).

The cadaveric dissections were performed at the Midas Rex Institute in Fort Worth, Texas, USA. Three adult cadaveric heads that were injected and formalin-fixed were used. Red and blue silicon was used for the injection of the arteries and veins, respectively.

Before proceeding to the dissection, a computed tomography (CT) was performed on the three heads, and the registration for neuronavigation was done. Datasets of the CT were as follows: tilt gantry 0°, matrix 512 × 512 pixels, resolution 0.46 mm/pixels, slice thickness 0.63 mm, as suggested [12]. Afterward, the heads were positioned to simulate the approach with the setup required for the neuronavigation. Dissections were performed under direct visualization through a ZEISS PENTERO microscope (Carl Zeiss, Oberkochen, Germany). A high-speed drill (Medtronic Midas Rex) was used for craniotomy and drilling, while microneurosurgical instruments were employed for the dissection of the neurovascular structures of the petroclival region. The navigation system (Medtronic S7) showed the coordinates of the important landmarks during the whole dissection.

Photographs were taken with a Nikon D7200 camera with a Micro Nikon 40 mm F 2.8 objective and annular flash. The camera was set up in the same way for all the images, using a 20 diaphragm, 100 shutter speed, 250 ISO, and 1/128 annular flash.

To obtain the surgical images, the following were used: TIVATO surgical microscope (Carl Zeiss, Oberkochen, Germany); two video adapters with a focal length of 125 mm; two Blackmagic Micro Cinema cameras: 1920 × 1080, 1080 p 24 fps (Blackmagic Design, Port Melbourne, Victoria, Australia); HDMI cable; and Demultiplexer software v. 1.0.98.

## 3. Results

### 3.1. Cadaveric Dissection

#### 3.1.1. Surgical Position and Bony Exposure

The heads were secured in a Mayfield head holder and placed in a lateral position, parallel to the floor. A reversed question mark preauricolar skin incision was utilized, having as landmarks the tragus and midpupillary line. An interfascial dissection was carried out to reduce the risk of damage to the frontal branch of the facial nerve. Subperiosteal dissection of the temporalis muscle allowed skeletonizing the squamosal part of the temporal bone and the posterior root of the zygoma.

#### 3.1.2. Craniotomy and Middle Fossa Peeling

A partial zygomatic osteotomy was carried out from the lateral margin of the orbit anteriorly to the temporomandibular joint posteriorly. Zygomatic osteotomy increased the surgical freedom, allowing a greater angled visualization beneath the temporal lobe, reducing the need for brain retraction. A frontotemporal craniotomy was then completed. The base of the craniotomy was parallel to the zygomatic arch and the floor of the middle fossa, just above the root of the zygoma and the external auditory meatus, with two-thirds anterior to and one-third posterior to the external auditory canal. The remaining part of the temporal squama was drilled down to the level of the floor of the middle fossa to improve the view further. Next, the dura mater was carefully dissected from the petrous temporal bone and elevated in a posterior-to-anterior and lateral-to-medial direction. At the level of the middle fossa, the periosteal dura was peeled cranially from the temporal dura. The middle meningeal artery (Mid. Men. A.) was cut at the level of foramen spinosum. This maneuver allowed for a better full exposure of the foramen ovale anteriorly in the surgical field. Care must be taken to perform an inter-dural dissection. The dura propria is separated from the connective tissue sheath of V3 and the lateral aspect of the Gasserian ganglion (GG) [13]. This maneuver enables relaxation of the dura covering the temporal lobe and improves its mobilization [14].

#### 3.1.3. Drilling of the Petrous Bone under Neuronavigation

**Figure 1:**
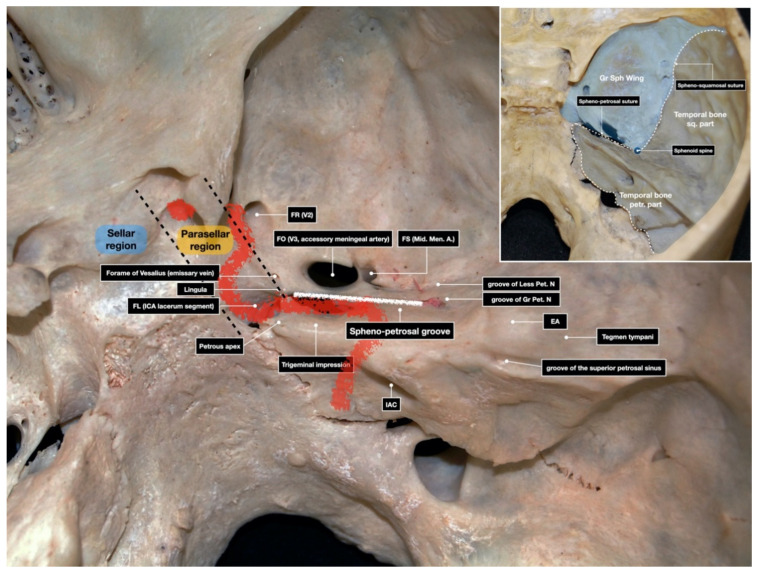
Main bony landmarks of the middle and posterior skull base. The red and white lines mark the course of the internal carotid artery and spheno-petrosal groove, respectively. EA: Eminentia Arcuata; FL: Foramen Lacerum; FO: Foramen Ovale; FR: Foramen Rotundum; Gr Sph. Wing: Greater Sphenoid Wing; Gr. Pet. N.: Great Petrosal Nerve; IAC: Internal Auditory Canal; ICA: Internal Carotid Artery; Less. Pet. N.: Lesser Petrosal Nerve; Mid. Men. A.: Middle Meningeal Artery; Temporal Bone sq. part: Squamosal part of the temporal bone.

The middle fossa floor was exposed, and the sphenopetrosal groove was easily identified. It is a landmark for the horizontal portion of the petrous carotid artery (Car. A.), similarly to the line passing through the foramen ovale and the foramen spinosum. The sphenopetrosal groove is a bony depression which is bounded by the trigeminal impression anteromedially, the emimentia arcuata (EA) posteromedially, the carotid canal inferiorly, and the internal auditory canal (IAC) inferolaterally (Figure 1).

The EA is another surgical landmark; it is a rounded bony elevation overlying the superior semicircular canal (SSC), the integrity of which is mandatory to preserve hearing. EA may be flat in 15% of cases [15].

**Figure 2:**
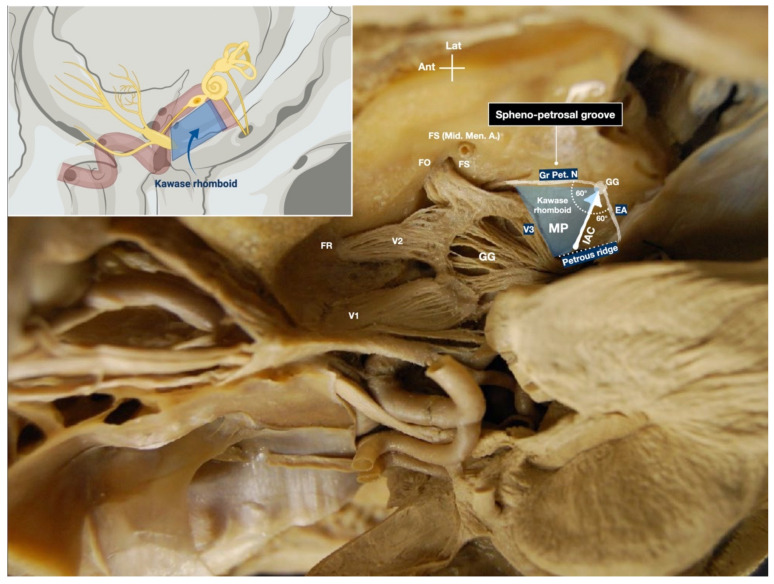
Exposure of the trigeminal nerve in the Merkel’s cave and main landmarks for the unroofing of the internal acoustic meatus and anterior petrosectomy. The sketch in the upper left side of the figure shows the Kawase rhomboid. Ant: Anterior; EA: Eminentia Arcuata; FO: Foramen Ovale; FR: Foramen Rotundum; GG: Gasserian Ganglion; Gr. Pet. N.: Great Petrosal Nerve; IAC: Internal Auditory Canal; Lat: Lateral; Mid. Men. A.: Middle Meningeal Artery; V1: Ophthalmic division of the trigeminal nerve; V2: Maxillary division of the trigeminal nerve; V3: Mandibular division of the trigeminal nerve.

The greater superficial petrosal nerve (Gr. Pet. N.) runs in the sphenopetrosal groove from the hiatus facialis to the foramen lacerum, where it joins the deep petrosal nerves to form the vidian nerve. Sometimes, it may need to be cut in order to avoid injury to the facial nerve during dural dissection [7]. At this step, the identification of the meatal plane is important, since it contributes to form the bony roof of the IAC. The meatal plane is bounded posteriorly by the EA, laterally by the Gr. Pet. N., anteromedially by trigeminal impression, and posteromedially by the petrous ridge. The same structures mark the limit of the drilling of the anterior petrosectomy (Figure 2). The anterior pyramidal bone is usually of a softer consistency than the middle ear bone, and this offers an important orientation during pyramid resection.

**Figure 3:**
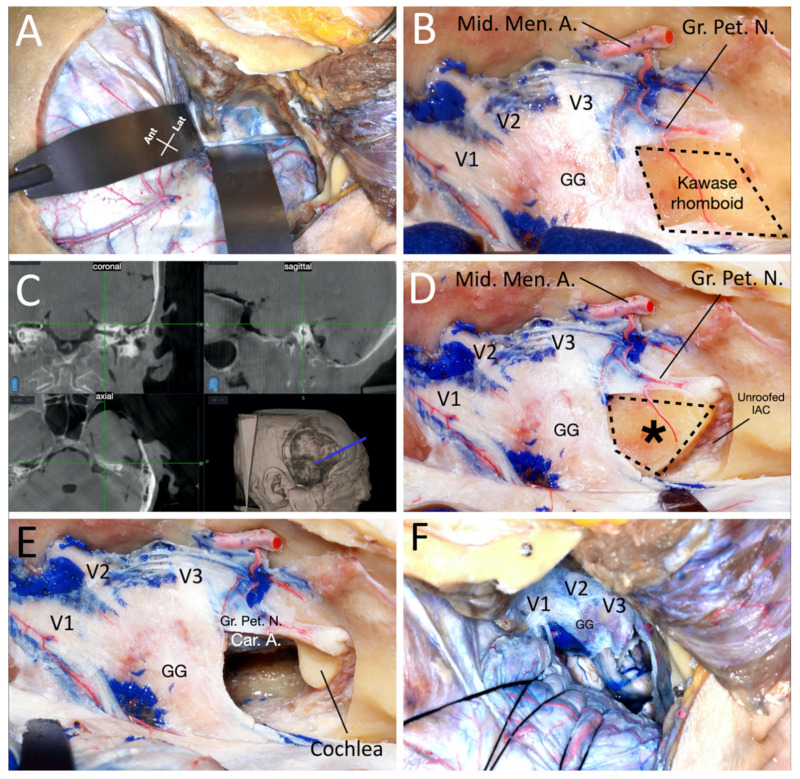
(**A**) Extradural exposure of the middle fossa. (**B**) Interdural skeletonization of the Gasserian ganglion and first, second, and third division of the trigeminal nerve. (**C**) Navigation guidance during the anterior petrosectomy. (**D**) Unroofing of the internal auditory canal. The asterisk marks the Kawase rhomboid. (**E**) Anterior petrosectomy. (**F**) Dura opening and with the exposure of the pons along with the root entry zone of the trigeminal nerve. Ant: Anterior; Car. A.: Internal Carotid Artery; GG: Gasserian Ganglion; Gr. Pet. N.: Great Petrosal Nerve; IAC: Internal Auditory Canal; Lat: Lateral; Mid. Men. A.: Middle Meningeal Artery; V1: Ophthalmic division of the trigeminal nerve; V2: Maxillary division of the trigeminal nerve; V3: Mandibular division of the trigeminal nerve.

**Figure 4:**
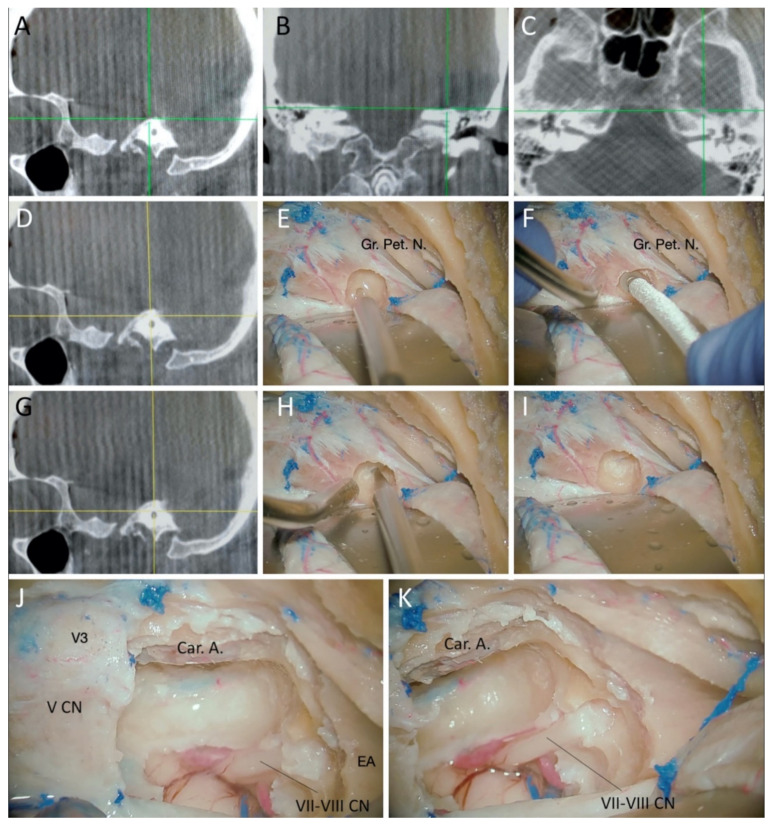
(**A**–**D**) Navigation screenshots showing the trajectory of the drilling towards the internal acoustic meatus in the sagittal (**A**,**D**), coronal (**B**), axial (**C**). (**E**,**F**) Tip of the probe marking continuously the position of the drilling of the bone between the greater petrosal nerve and the eminentia arcuata in the navigation screen. (**G**) Navigation screenshots in sagittal view, revealed as the drilling approximates the dura of the internal auditory canal. (**H**,**I**) Tip of the probe marking the dura of the internal auditory canal. (**J**) Final exposure of the approach, with the following limits: the posterior margin of V3, medial margin of the internal carotid artery, and anterior margin of the eminentia arcuata. The dura of the internal auditory canal was opened to expose the VII-VIII complex. (**K**) Final view of the intradural exposure of the approach. Car. A.: Internal carotid artery; EA: Eminentia Arcuata; Gr. Pet. N.: Great Petrosal Nerve; IAC: Internal Auditory Canal; V CN: Trigeminal cranial nerve; V3: Mandibular division of the trigeminal nerve; VII-VIII CN: facial and vestibulocochlear cranial nerves.

The bone was drilled out with a high-speed diamond drill under navigation guidance. The drill was connected to the neuronavigation system. The whole drilling was performed with strict control of our position. We used the coronal, sagittal, and axial projections to gain orientation in relation to the important and non-visible landmarks: IAC, Car. A., SSC, and the cochlea (Figure 3 and Figure 4). With continuous irrigation, the drilling was started between the greater petrosal nerve and the EA under constant multiplanar navigation as the drilling went further towards the IAC (Figure 3). This maneuver allows for the unroofing of the IAC and exposes the underlying dura (Figure 3D).

Bone removal continued from medial to lateral, following the dura of the IAC (Figure 3 and Figure 4). The cochlea lies anterior to the fundus of the IAC, below the geniculate ganglion. It is important to avoid fenestration of the cochlea to preserve the hearing; the navigation allowed us to identify the position of this structure during the drilling for its preservation with maximal exposure. Once the drilling of the petrous pyramid was concluded, the Car. A. and dura of the posterior fossa were exposed. The dura of the IAC and the posterior fossa were opened, revealing the lateral aspect of the pons, roots of the trigeminal nerve, VII-VIII complex, and basilar artery at the anterior part of the exposure (Figure 3F). A T-shaped cut was performed over the temporal dura and anterior to the superior petrosal sinus. The latter was then ligated, exposing the tentorium. After identifying the trochlear nerve, the tentorium was divided until the tentorial notch. The exposure of the lateral part of the upper and middle clivus and petroclival region was maximized, removing the bone under the trigeminal ganglion to include the petrous apex.

### 3.2. Surgical Case

**Figure 5:**
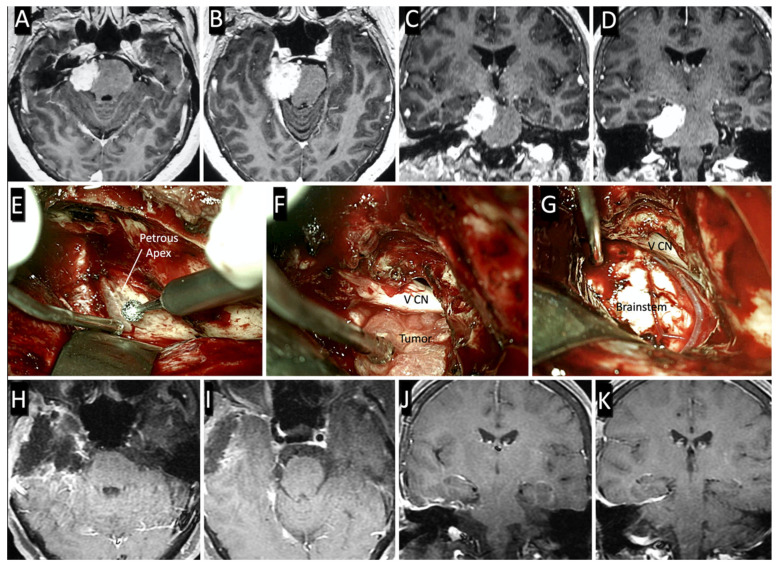
Preoperative axial (**A**,**B**) and coronal (**C**,**D**) T1-weighed contrast-enhanced MRI showing the petroclival meningioma. (**E**–**G**) Main steps of the anterior petrosal approach. (**E**) Peeling of the middle fossa dura and exposure of the petrous apex. (**F**) Intradural tumor exposure and initial debulking. (**G**) Surgical field after tumor resection and visualization of the lateral aspect of the pons. Postoperative axial (**H**,**I**) and coronal (**J**,**K**) contrast-enhanced T1-weighed MRI showing the gross total resection of the tumor (Simpson grade I).

A 52-year-old woman suffered from a headache. Neurological examination was unremarkable. Contrast-enhanced MRI scans revealed a right petroclival meningioma. The tumor caused brainstem compression and moderate midline shift. The patient underwent the anterior petrosal approach with neuronavigation guidance. Once the approach was completed, the root entry zone of the trigeminal nerve and the tumor were exposed. Note that the tumor was medial to the trigeminal nerve, as by design in “true” petroclival meningioma. An anterior petrosal approach allowed for wide exposure of the upper petroclival area, and a gross total removal of the meningioma was achieved (Simpson grade I) with no complications. Postoperative MRI confirmed the total resection of the tumor (Figure 5).

The postoperative course was favorable without neurological deficits, and the patient was discharged seven days after surgery. Pathology revealed a meningothelial meningioma (WHO grade I).

## 4. Discussion

### 4.1. Previous Concepts

The extended middle fossa approach was described for the first time in 1975 by Bochenek and Kukwa [16]. The “extension” involves the unroofing of the IAC, and the exposure of the cerebellopontine angle from the middle fossa perspective. Kawase and colleagues suggested an anterior transpetrosal approach, also encompassing the cutting of the tentorium, to treat basilar tip aneurysms, and petroclival and sphenopetroclival tumors [7,11,17]. This subtemporal anterior transpetrosal transtentorial approach was later popularized as the “Kawase approach”. The Kawase approach provides a wide exposure of the upper clivus and the porus trigeminus. The limits of the so-called Kawase rhomboid are the greater superficial petrosal nerve laterally, petrous ridge medially, mandibular division of the trigeminal nerve (V3) anteriorly, IAC, and anteromedial margin of the EA posteriorly. Several modifications of the Kawase approach have been reported [1].

The anterior petrosectomy approach is today widely used for sphenopetroclival meningiomas, dumbbell-shaped trigeminal schwannomas, vestibular schwannoma, basilar apex, and mid-basilar trunk aneurysms, and ventral and lateral pontine cavernous malformations [18].

### 4.2. Anatomic Details and Neuronavigation

Intraoperative neuronavigation is universally considered today as a helpful tool for neurosurgery in light of its high accuracy [19,20]. Of note, this accuracy is even greater when dealing with skull base structures given the lack of brain shift [21]. Literature reports several morphometric studies aimed at the identification of critical surgical landmarks for cranial base surgery [22,23]. While the rate of anatomical variations of the petroclival area is relatively low, it cannot be considered negligible [24]. That is the main reason why the routine use of neuronavigation is recommended for an intraoperative constant check of the neurovascular structures, thus decreasing the risk of iatrogenic injuries. Furthermore, the navigation aids in tailoring the approach to specific anatomical features of the patient. Literature about the accuracy of neuronavigation, particularly in regard to temporal bone surgery, is relatively poor. This is because the petrous apex and the sphenopetroclival regions have a series of bony landmarks which facilitate the dissection and localization of the structures regardless of the use of navigation [12,25]. The morphometric analyses and anatomical studies of the petrous apex described the relationships between the critical structures of this region, namely the cochlea, the IAC, and the bony labyrinth. To localize the Car. A., Dolenc described the posterolateral or “Glasscock” triangle, which is limited by the Gr. Pet. N. laterally, mandibular nerve medially, and posterior loop of the petrosal Car. A. posteriorly. The petrous segment of the Car. A. lies within this triangle [26]. The Car. A. is found in the area beneath the greater superficial petrosal nerve, although Dew et al. reported that the course of the nerve over the artery is extremely variable [12]. In exposing the horizontal petrous Car. A, no definite landmarks are available to localize the boundaries of the cochlea. In fact, only a few millimeters of bone separate them. A further limitation in the localization of the horizontal petrous Car. A., regarding the cochlea during preoperative planning, comes from the fact that they are not on the same slice on the standard axial and coronal CT scan.

In a detailed morphometric study on the extradural middle fossa, Day et al. reported that the cochlea is located within the lateral portion of the premeatal triangle, which is bounded by the geniculate ganglion, Car. A. genu, and IAC [22]. They also measured the distance between the cochlear apex and the genu of the petrous Car. A., defining it as 2.8 mm ± 1.3 [22].

Tedeschi et al. suggested the localization of the cochlea be in the angle formed by the Gr. Pet. N and the IAC [27]. They also suggested a simple method to mark the course of the IAC on the middle fossa floor: It corresponds to the bisect of the angle between the Gr. Pet. N and the IAC, the latter measuring 120° [27]. Miller et al. described how the cochlea lies mostly beneath the geniculate ganglion [28]. Kim et al. defined the Gr. Pet.N.-Car. A. point and the IAC line to identify the cochlear line and the IAC point to mark the anteromedial perimeter of the cochlea [29]. Steiger and colleagues found the distance between the top of the EA and the axis of the IAC to be 3 to 5 mm, while the length between the anteromedial tip of the petrous ridge and anterior wall of the IAC ranges from 13 to 18 mm [30]. Wang et al. found a high variability rate of the modiolar axis and turns of the cochlea with regards to the adjacent structures [31]. That is the reason why we believe that the neuronavigation is helpful at this point of the approach, once V3 and Gr. Pet. N. are exposed, to assess the exact location of the IAC and cochlea, avoiding blind drilling of the petrous bone.

Komune and colleagues from the Rhoton’s group highlighted that, while being relatively limited, the anatomical variability of the landmarks of the middle fossa approach is non-negligible [17]. For this reason, careful preoperative planning and the intraoperative implementation of neuronavigation make it possible to tailor the anterior petrosal approach to the patient’s specific anatomical features.

The advantages of using neuronavigation are, for instance, structure identification, route planning, visualization of vital structures deep inside a lesion, and better orientation to patient anatomy and its individual variability [32,33]. Given the extremely limited size of the structures located within the temporal bone, the minimal accuracy threshold required for neuronavigation in temporal bone surgery consists of an error margin that must be less than 0.5 mm. The use of image-guidance in temporal bone surgery was reported to bring neither risk of complications for the approach nor extra surgical operating time [32].

### 4.3. Advantages and Disadvantages of Anterior Petrosectomy

Among the major strengths of the anterior petrosectomy versus transcochlear approach, Danner et al. reported a low risk of hearing loss, facial nerve palsy, and vestibulopathy in a wide series of pathology affecting the petroclival region and the anterior cerebellopontine angle [34].

However, the anterior transpetrosal route is not free from the jeopardy of significant approach-related morbidity [22]. Possible complications are, first, traction injury of the Gr. Pet.N., causing facial nerve palsy or iatrogenic dry eye syndrome, and second, injury or thrombosis of the vein of Labbé with temporal lobe infarction.

Based on the easiness of their identification during the approach, the neurovascular structures that can be potentially injured may be listed in two groups: visible elements, involving the Mid. Men. A., V3, GG, and Gr. Pet. N.; and the non-visible ones, namely the cochlea, SSC, and IAC. While the elements of the first group are generally preserved if the approach is correctly performed from the technical standpoint, those of the second one may be more easily injured during the drilling of the meatal plane. Among these, the Car. A. has a good anatomical landmark, namely the Gr. Pet. N., differently from the cochlea and IAC. For all these reasons, we believe that deep anatomical knowledge is mandatory, along with the use of neuronavigation to perform the anterior petrosectomy approach safely.

## 5. Conclusions

Neuronavigation during the anterior petrosectomy was successfully performed in all the specimens, and also the surgical case demonstrated its clinical effectiveness. It achieved an adequate exposure of the surgical target, at the same time preserving the integrity of visible and non-visible elements.

The drilling of the petrous apex during the anterior petrosectomy includes the possibility of damage to the Car. A cochlea, Gr. Petr.N., superior semicircular canal, and contents of the IAC. Deep anatomical knowledge and tridimensional mental figuring of these structures, combined with the use of a neuronavigation, are the key factors to reduce the risk of complications during the anterior petrosectomy approach.

Further evidence on larger anatomical and clinical studies is necessary for a proof of concept about the effectiveness of neuronavigation during temporal bone surgery.

## Data Availability

Data are contained within the article.
